# Multiple Solutions Starting from Real Shaped Beams in Equispaced Linear Arrays

**DOI:** 10.3390/s21010062

**Published:** 2020-12-24

**Authors:** Aarón Ángel Salas-Sánchez, Camilo López-Castro, Paolo Rocca, Juan Antonio Rodríguez-González, María Elena López-Martín, Francisco José Ares-Pena

**Affiliations:** 1 ELEDIA@UniTN, Department of Information Engineering and Computer Science (DISI), University of Trento, 38122 Trento, Italy; aaronangel.salas@usc.es (A.Á.S.-S.); paolo.rocca@unitn.it (P.R.); 2 CRETUS Institute, Department of Applied Physics, University of Santiago de Compostela, 15782 Santiago de Compostela, Spain; camilo.lopez.castro@rai.usc.es (C.L.-C.); ja.rodriguez@usc.es (J.A.R.-G.); 3 CRETUS Institute, Department of Morphological Sciences, University of Santiago de Compostela, 15782 Santiago de Compostela, Spain; melena.lopez.martin@usc.es

**Keywords:** bandwidth, equispaced linear arrays, far field patterns, multiplicity of solutions, shaped-beam patterns, tolerance analysis, circular footprints, elliptical footprints

## Abstract

In the present work, the theoretical basis of the multiplicity of solutions obtained from an initial real symmetric distribution is derived. This initial solution is devoted to generating an equivalent pure real shaped-beam pattern for a concrete synthesis scenario. However, these new solutions are not based on real symmetric distributions; hence, not based on the generation of pure real patterns. The bandwidth performances and tolerance to errors provided by the multiple solutions in the array design are analyzed by considering different architectures, also including mutual coupling models and element factor expressions due to accuracy purposes. In addition, a technique to obtain efficient linear arrays by designing resonant structures is addressed. Examples involving both standard linear arrays of half-wavelength cylindrical dipoles and resonant linear arrays generating flat-top beam patterns are reported and discussed. Additionally, an extension to planar arrays performed by means of a generalisation of the Baklanov transformation through collapsed distribution techniques inspired in the well-known method devised by Tseng and Cheng is performed. In such a way, an analysis of the quality of solutions for generating circular and elliptical footprints with controlled both SLL and ripple which are highly interesting in the framework of space vehicle applications.

## 1. Introduction

Shaped-beam array patterns are widely-used for antenna and radar applications. More precisely, they represent a very interesting solution in satellite application for providing a uniform coverage of a certain region on the surface of the Earth or similar. Thus, many techniques have been attracting the attention of many researchers since the introduction in 1947 by Woodward [1] of the classical problem of generating a shaped pattern from an equispaced array. A conceptually simple—i.e., computationally inexpensive—methodology, based on a polynomial representation of the radiation power pattern (by means of the array factor) has been presented in [2]. This approach effectively controls the ripple level provided in the shaped region as well as the topography of the side lobes by obtaining a far field radiation pattern with deep nulls in the unshaped region (the region towards the main beam). At the same time, a multiplicity of solutions generating the same far field radiation pattern can be directly derived from this formulation.

By analyzing the state of the art on shaped-beam in linear array pattern synthesis works as [3] can be referred where arbitrary shaped-beam patterns are generated by means of sub-array clustering solutions. Here, an optimal trade-off between the complexity of the array structure and the matching of a reference pattern is exploited. Another interesting alternative [4] proposes a superposition of scanned sum patterns for the shaped-beam pattern generation. With regard to the introduction of multiple sets of solutions within the array pattern synthesis problem, alternative methodologies from recent literature can be highlighted [5,6,7]. These works are mainly focused on solving mask-constrained problems through arbitrary array layouts and thus they can effectively deal with an arbitrary spacing between the elements. Otherwise, although some interesting attempts with the unit circle representation from aperiodic arrays have been made in [8], the methods inspired by Schelkunoff [9] (in the basis of the present work) certainly do not allow a direct management of non-uniformly spaced linear arrays.

The methodology based on dealing with equispaced linear arrays are directly linked to the generation of radiation far-field patterns showing deep nulls. There are of course, alternative methods as [10] that can be used as input of the procedure, but we have chosen the Orchard–Elliott–Stern method [11], a powerful technique that uses the physical principles of adjusting zeros in the array pattern polynomial. Even, in light of the comments made by some authors arguing about the performance of the Orchard–Elliott–Stern method, it was shown that this procedure can deal with extreme ripple conditions as well as real symmetric and asymmetric far-field radiation patterns [12]. As general comment for supporting the application of the Orchard–Elliot–Stern method one can state that this procedure represents a fast, cheap and efficient numerical method for obtaining a certain root positioning to generate a concrete far field radiation pattern on equispaced linear arrays. Then, considering that the root positions given by the specifications in the synthesis problem (ripple, beamwidth, falloff from the shaped region, and side lobe levels) are unique and in light that these roots generate far-field patterns exhibiting deep nulls, one can guarantee the performance of this technique at the level of the state-of-the-art in the antenna array pattern synthesis. In summary, one can understand the array pattern synthesis as a framework of cleverly positioning the zeros of the polynomial for a certain antenna array solution.

On the other hand, as is well known for these Schelkunoff-based techniques, the multiplicity of their solutions in case of analyzing a complex shaped-beam pattern is 2^2M^ [11], where 2M is the number of nulls which has to be filled by the synthesis procedure. Here, a symmetric number of elements in the array and as a consequence a symmetric number of filled nulls have been assumed without any loss of generality.

Regarding pure real shaped-beam patterns, Elliott and Stern [2]—inspired by the technique introduced by Woodward [1]—demonstrated in 1984 that they can be generated by means a displacement of the roots in pairs at the same angular position in the Schelkunoff unit circle. In such a way, the realisation of pure real patterns can be assured, including asymmetric shaped-beam patterns, and approaches as the proper Woodward technique [1] or the extensions of Orchard–Elliott–Stern synthesis method [13] have proven this performance. In particular, the reader can consider the synthesis of an asymmetric pure-real pattern to obtain one real symmetric set of excitations in [13] (by means of a generalization of the symmetric procedure devised by Kim and Elliott [14]).

In the present work, a methodology for introducing multiple distributions generated from this initial constrained pure real pattern case (equivalent to the classical complex pattern case) is addressed and fully characterized. Thus, an enlargement of the number of valid solutions to the synthesis problem and, therefore, a new path for analyzing the synthesis problem in shaped-beam patterns—envisaged in [15]—is explored and its theoretical bases are developed. Moreover, as restricted studies about the nature of these resulting multiple excitation distributions have not been developed in the literature until now and just a preliminary study confirming this variability and addressing limited bandwidth studies [16], the theoretical peculiarities of the method regarding these different types of solutions are here discussed. In fact, the number of solutions produced in the synthesis of an initial pure real far field pattern—envisaged in [15] as 3^2M^ and confirmed in [16]—is now fully addressed and characterized from a theoretical point of view.

An interesting feature of exploiting this new multiplicity of solutions is the facilitation of the physical realisation of the array. In this manner, the chance to select an adequate solution with regard to certain feeding network scenario (end-fed, centre-fed, as well as corporate fed arrays) is highly interesting. As a drawback, a slightly enlargement of the number of elements of the array has to be expected in comparison to the standard complex pattern case, if we want to keep the same number of ripples in the shaped region. Attending the quality of the results offered by this new procedure, both bandwidth and tolerance analyses of the entire multiplicity of solutions (the classical multiplicity and the one devised in the present work) are here proposed. In such a way, the potentials of the new set of solutions can be understood while their quality is evaluated within the framework of the application in linear array synthesis. Additionally, an extension to both circular and elliptical footprint patterns by means of a generalized procedure of the well-known Tseng and Cheng methodology [17] are applied. In such a way, the performance of the different solutions to produce extended 2-D far field patterns will add impact to the present study, motivating the provided development for space vehicle applications in order to facilitate both practical realisation and power consumption.

Regarding bandwidth, studies on equally spaced linear arrays synthesizing flat-top beam patterns have been previously faced in [18,19]. In both referred works, classical approaches to these multiplicity problems have been devoted to perform a bandwidth study of the different set of solutions. More concretely, in [18], an example of a collinear cylindrical dipole array case has been analyzed, while an analysis involving linear arrays of parallel cylindrical dipoles has been reported in [19]. These approaches have addressed excitation distributions which generate complex far field patterns and the special case of a unique distribution generating a pure real radiation far field patterns (more concretely, the pure real distribution of [14]). Additionally, it is worth mentioning that mutual coupling impedance terms among cylindrical dipoles of common length (λ_0_/2) have been modelled, where λ_0_ is the wavelength at the design frequency. To this aim, a formulation for modelling mutual coupling effects developed by Hansen [20] has been implemented. This strategy is no longer applicable to determine embedded impedance terms of an array with variability in the lengths of its elements. More precisely, the expressions from the work of Hansen for the mutual impedance cannot manage different lengths of the slender dipoles. An attempt to deal with a less restricted formulation has been addressed in [16], but these methods also present limitations in terms of elements sizes (changes in the lengths of the dipoles 1.3≤kl≤1.7 and element radii 0.0016≤a/λ≤0.0100 were considered). To fill this gap, improved expressions of mutual coupling based on standard methods [21] have been integrated into the methodology here depicted. In this manner, a management of different lengths of the elements of the array can be guaranteed.

With regard to tolerance analysis, examples also devoted to report the performance of a classical multiplicity of solutions by generating the same shaped-beam pattern, have been developed in [22]. In this work, statistical studies about the sensitivity of the performance to errors in element positions, excitation phases or amplitudes have been conducted. As limitation to highlight, the work was performed without introducing a model for the coupling effects neither taking care about the radiation field of each one of the dipoles which define the array (i.e., their element factors). So, for understanding the potentials of the multiplicity of solutions created by means of a distribution generating a pure real shaped-beam pattern, a tolerance analysis is here proposed. As it was already mentioned, the lack of modelling mutual coupling effects, as well as element factors, in previous strategies [22] adds motivation to conduct this specific study.

Finally, from the planar arrays scenario, the potentials of selecting a more convenient solution within the multiplicity are shown. In this manner, the generalisation of the Baklanov transformation [23] illustrates the behavior of the solutions in different test cases with different number of elements in the two directions of the planar array by linking the pattern to a polynomial of one variable as proposed in [24]. Therefore, a theoretical discussion regarding the orders of this polynomial has been discussed by López-Castro [25] and a compact expression to deal with both odd and even p and q parameters of the general transform has been derived in the present work. So, the evaluation of the quality of the solutions obtained by means of the multiplicity is reported through the variability of the excitation currents in the synthesized planar array.

In order to illustrate the method, 10-element and 12-element linear arrays have been considered within the pure real shaped-beam pattern synthesis, by completing the standard discussion of [14]. This work analyses a shaped-beam pattern synthesis problem from the point of view of a 12-element linear array in terms of a unique pure real excitation distribution by means of this particular linear array arrangement. So, in the present work, this pure real shaped-beam pattern is used to generate, discuss, and analyse one part of the innovative multiplicity of solutions here proposed. This is due to, under the philosophy of [14], the main concern is guaranteeing the same number of ripples in the shaped region. Here, alternatively, we are also exploring the possibility of keeping the same number of elements as in the initial complex pattern case (10 radiating elements). In this case, the cost to pay is to reduce the number of ripples in the shaped region to the half and consequently to decrease the number of multiple solutions produced by the method. At the same time, linear arrays of 10 elements have been selected for generating the complex shaped-beam pattern multiplicity (i.e., the standard/known method) in order to provide an equivalent case. The initial distributions of the three cases have been obtained by means of the Orchard–Elliott–Stern technique [11]. Attending the model of each radiating element, linear arrays with default λ_0_/2 cylindrical dipoles and also linear arrays with optimized dipole lengths (devoted to create improved resonant structures) have been analyzed. In both cases, array architectures in absence and in presence of a ground plane—placed at a distance of λ_0_/4—have been simulated. To establish a notation and for clarification purposes, the denomination of complex pattern case (CPC) and real pattern case (RPC) has been adopted in the paper. The first one (CPC) is referred to the case of a multiple set of solutions obtained from an initial distribution that generates the complex far field pattern associated to the problem. Otherwise, the RPC is the reference to the multiplicity of solutions obtained from an initial distribution constrained to generate an equivalent pure real pattern synthesis (a pure real shaped-beam pattern with the same number of ripples). To differentiate between the two scenarios led by an initial real far field pattern the number of elements of the array (10 and 12) will be made explicit on each case.

## 2. Materials and Methods

Let us consider a linear array of 2*N* equispaced dipoles distributed along the *z*-axis, for which the array factor is led by the expression [21]:

(1) $$F\left(\theta \right)=\;\overset{2N}{\underset{n=1}{\sum }}f_{n}\left(\theta \right)I_{n}e^{jk\left(n-1\right)d\cos\theta },$$

where $f_{n}\left(\theta \right)$ is the radiation pattern of each one of the elements (in the case of isotropic radiators it would be 1), $I_{n}$ is the complex excitation current of the *n*-th element, *k* is the wavenumber and *d* is the spacing between elements.

### 2.1. Multiplicity Considerations with an Initial Pure Real Far Field Pattern

Once the excitations in (1) are determined by means of the Orchard–Elliott–Stern technique [11] or Kim–Elliott procedure [14], we can factorize the obtained array factor and represent it over the Schelkunoff unit circle.

Accordingly, if we analyse the case of an equispaced array of isotropic elements given by (1) it is well known (by means of the fundamental theorem of algebra) that the roots of the polynomial can be factored as

(2) $$\overset{2N}{\underset{n=1}{\sum }}\frac{I_{n}}{I_{2N}}\omega^{n-1}=\overset{2N-1}{\underset{i=1}{\prod }}\left(\omega -\omega_{i}\right)=0,$$

where 2*N* − 1 is the number of roots of the polynomial $\left(\omega_{i}\right)$ and, by means of the change ψ=kdcosθ, and therefore $\omega =e^{j\psi }=e^{jkd\cos\theta }.$ These roots can be expressed by $\omega_{i}=e^{\left(a_{i}+jb_{i}\right)},$ which are the group of complex elements to be represented in the Schelkunoff unit circle. Then, multiple sets of different solutions will be obtained by altering the positions of the roots within the Schelkunoff unit circle representation of the initial set. In such a way, the same normalized amplitude of the far-field pattern is produced. In the case of interest for the present work, a pure-real shaped-beam pattern will certainly generate equivalent far field patterns of different natures.

So, by altering the part of the set which lies off the Schelkunoff unit circle, equivalent solutions in terms of the far field pattern are produced. More precisely, as it has been pointed out in [14,15], if we alter the sign of $a_{n}$ linked to any of the roots off the unit circle, the magnitude of the obtained radiation power pattern will describe exactly the same function as the initial case. In the complex case, the number of different solutions which can be generated are $2^{Q_{c}}$ for $Q_{c}$ roots off the unit circle [11], while $3^{Q_{r}/2}$ is the number of different solutions offered in the real case being $Q_{r}$ the necessary number of roots off the unit circle for generating a pure real pattern [15].

**Figure 1:**
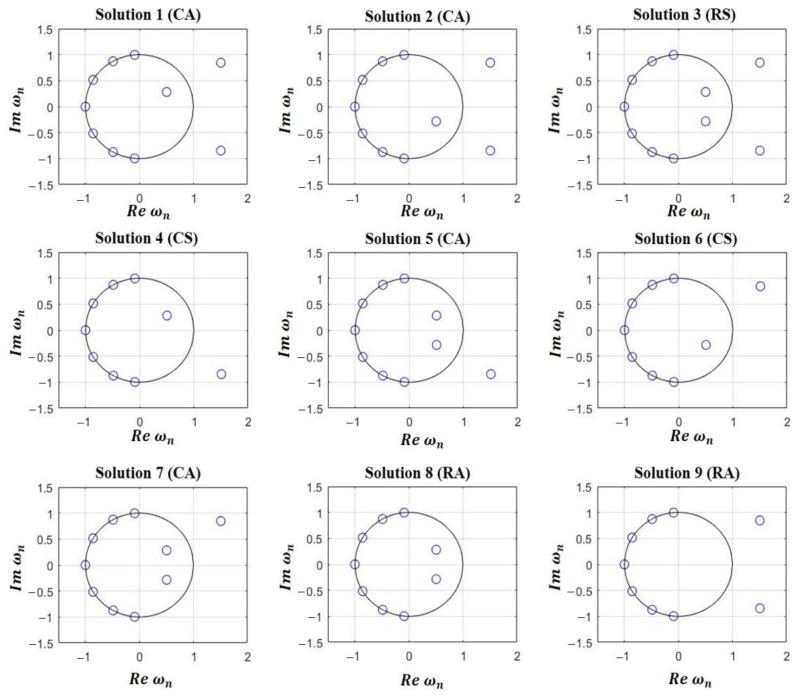
The nine possibilities about root positions corresponding to a 12-element linear array (sketched in Figure 3) designed to produce flat-top beam with two filled nulls (M=1), a ripple of ±0.5 dB and a sidelobe level of −20 dB. In terms of nature: Solution **3** is real symmetric (RS), Solutions **8** and **9** are real asymmetric (RA), Solutions **4** and **6** are complex symmetric (CS), and Solutions **1**, **2**, **5**, and **7** are complex asymmetric (CA).

**Figure 2:**
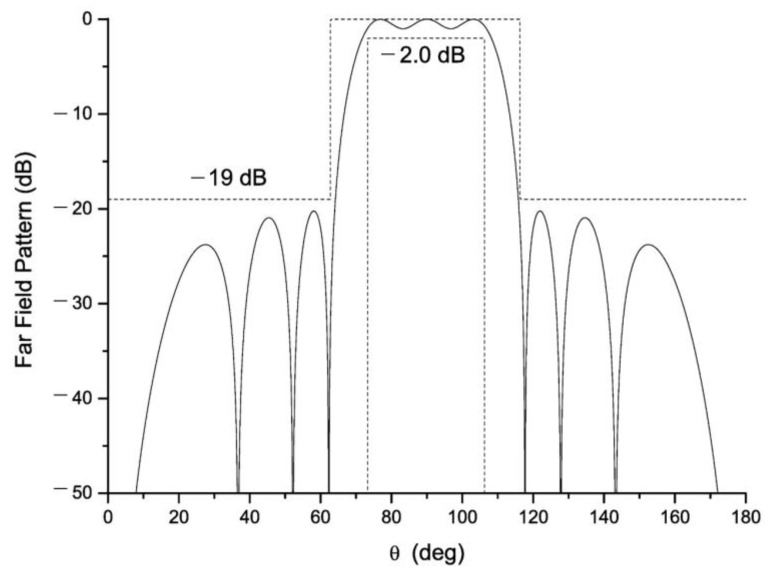
Solid curve: A symmetric flat-top beam obtained by the Kim–Elliott procedure [14] with 2 filled nulls (*M* = 1), ±0.5 dB of ripple, and a maximum sidelobe level of −20 dB. Dashed curves: Mask defining the limits to meet by the pattern for a successful sample in the tolerance analysis.

**Figure 3:**
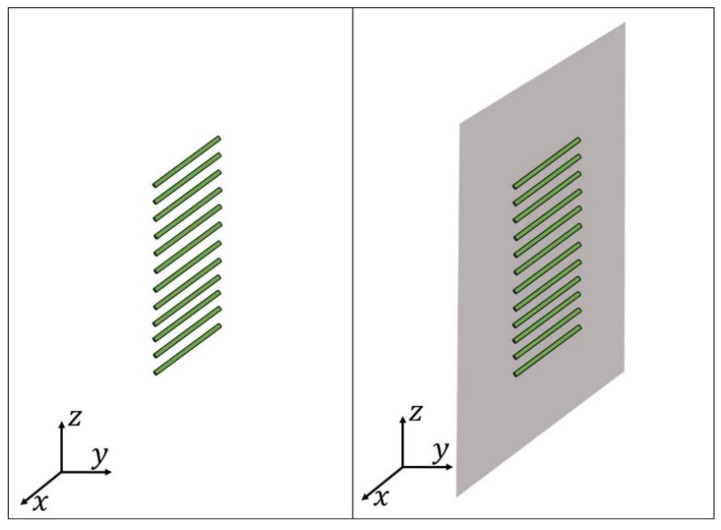
Sketch of the proposed 12-element linear array for the real pattern case (RPC): in absence of ground plane (**left**), in presence of ground plane (**right**).

An example of this multiplicity of solutions is shown in Figure 1, where the 9 possibilities (non-repeated solutions) for generating the same shaped-beam pattern with two filled nulls, a SLL of −20 dB and a ripple of ±0.5 dB (Figure 2, solid curve) are distinguished in the case of a linear array with 12 elements (Figure 3). It is important to have in mind that, as it has been shown in [14], it is necessary to have a couple of roots (in complementary positions off the Schelkunoff unit circle, see Appendix A) for each null to force the generation of a pure-real radiation pattern (i.e., $Q_{r}=2Q_{c}$) and in this manner, obtain a pattern similar to the CPC. Thus, while in the CPC the number of filled nulls 2M is equal to $Q_{c},$ for the RPC $2M=Q_{r}/2$ filled nulls are mandatory. This means that 2M more elements are required for synthesizing a far field pattern with the same number of ripples.

In a deep and general analysis about the nature of each one of the solutions for the RPC (in a similar way as the depicted in [14] for the CPC), if we have an equispaced linear array of 2*N* elements and it is desired to produce a symmetric shaped beam with 2*M* filled nulls (so 4*M* roots displace radially off the unit circle), then

(3) $$F\left(\omega \right)=I_{2N}\left(\omega +1\right)\cdot \overset{N-1-M}{\underset{i=M+1}{\prod }}\left[\omega^{2}-\left(2\cos b_{i}\right)\omega +1\right]\;\;\;\;\;\;\;\;\;\;\;\cdot \overset{M}{\underset{i=1}{\prod }}\left[\omega^{2}-\left(e^{a_{1,i}+jb_{i}}+e^{a_{1,i}^{\prime }-jb_{i}}\right)\omega +e^{a_{1,i}+a_{1,i}^{\prime }}\right]\;\;\;\;\;\;\;\;\;\;\cdot \left[\omega^{2}-\left(e^{a_{2,i}+jb_{i}}+e^{a_{2,i}^{\prime }-jb_{i}}\right)\omega +e^{a_{2,i}+a_{2,i}^{\prime }}\right],$$

which is a polynomial with complex (C) or real (R), and symmetric (S), or asymmetric (A) coefficients, depending on the relations between the signs of the four $a_{1,i},\;{a^{\prime }}_{1,i},\;a_{2,1}$ and $a_{2,1}^{\prime }$ terms. More precisely, it is easy to understand that by exploiting each one of the possible combinations of the parameters $\left(\pm \left|a_{1,i}\right|,\;\pm \left|a_{1,i}^{\prime }\right|,\;\pm \left|a_{2,i}\right|,\;\pm \left|a_{2,i}^{\prime }\right|\right),$ $2^{4M}$ solutions can be generated. Otherwise, in contrast with the behavior analyzed in [14] for the general complex case, some different roots sign combinations would generate exactly the same polynomial coefficients. So, for example, if one checks the case in study having 2*M* = 2 (i.e., *M* = 1) filled nulls, it is easy to understand that 4 solutions would lead to an exactly equal symmetric set of coefficients (Figure 1, Solution 3), whose root groups can be expressed as $+\left|a_{1,i}\right|,\;-\left|a_{1,i}^{\prime }\right|,\;+\left|a_{2,i}\right|,\;-\left|a_{2,i}^{\prime }\right|$. On other hand, 4 solutions with RA coefficients only generate two different set of coefficients (Figure 1, Solutions 8 and 9) which can be referred as $+\left|a_{1,i}\right|,\;+\left|a_{1,i}^{\prime }\right|,\;+\left|a_{2,i}\right|,\;+\left|a_{2,i}^{\prime }\right|$ and $-\left|a_{1,i}\right|,\;-\left|a_{1,i}^{\prime }\right|,\;-\left|a_{2,i}\right|,\;-\left|a_{2,i}^{\prime }\right|.$ Other 4 solutions with CS terms also produce just two independent solutions (Figure 1, Solutions 4 and 6), and in this case the roots can be expressed as $+\left|a_{1,i}\right|,\;+\left|a_{1,i}^{\prime }\right|,\;-\left|a_{2,i}\right|,\;-\left|a_{2,i}^{\prime }\right|$ and $-\left|a_{1,i}\right|,\;-\left|a_{1,i}^{\prime }\right|,\;+\left|a_{2,i}\right|,\;+\left|a_{2,i}^{\prime }\right|.$ Finally, the 4 related with CA coefficients have no chance to generate the same roots positions (Figure 1, Solutions 1, 2, 5, and 7), so they can be expressed as $-\left|a_{1,i}\right|,\;+\left|a_{1,i}^{\prime }\right|,\;+\left|a_{2,i}\right|,\;+\left|a_{2,i}^{\prime }\right|,$ $+\left|a_{1,i}\right|,\;-\left|a_{1,i}^{\prime }\right|,\;+\left|a_{2,i}\right|,\;+\left|a_{2,i}^{\prime }\right|,$ $+\left|a_{1,i}\right|,\;+\left|a_{1,i}^{\prime }\right|,\;-\left|a_{2,i}\right|,\;+\left|a_{2,i}^{\prime }\right|$ and $+\left|a_{1,i}\right|,\;+\left|a_{1,i}^{\prime }\right|,\;+\left|a_{2,i}\right|,\;-\left|a_{2,i}^{\prime }\right|.$ Another way to confirm the number of independent solutions in this problem is to understand that for the same filled null (at a certain angular position in the far field pattern), we necessarily will have 3 independent options. So, these options are: two paired roots, two double roots outside or two double roots inside the unit circle. Therefore, the total number of independent solutions is easily determined as 3^2*M*^; that is, 9 for the present example with *M* = 1. The reader can find in the Appendix A concrete details regarding the analytical expression of the far field pattern depending on the nature of this combination between roots.

So, generally, we have one arrangement of the roots for producing symmetric real coefficients (all the roots are paired in the Schelkunoff unit circle), 2*^M^* sets for no symmetrical real solutions (all the root pairs are double roots with symmetry along the horizontal axis of the unit circle representation, it is, $a_{2,i}^{\prime }=a_{2,i}=a_{1,i}^{\prime }=a_{1,i},$ 2^M^ sets of symmetrical complex solutions (all the root pairs are double roots with anti-symmetry with respect to the horizontal axis of the unit circle representation, it is, $a_{2,i}^{\prime }=-a_{2,i}$ and $a_{1,i}^{\prime }=-a_{1,i}$ with $a_{2,i}^{\prime }=a_{1,i}$ and the rest of the cases, namely, $3^{2M}-\left(1+2^{M+1}\right)$ coefficient sets, will represent the asymmetrical complex solutions.

To synthesize an equivalent pattern by means of the same number of elements, a distribution with just one couple of roots out of the Schelkunoff unit circle (at the same angular position) is necessary. So, in the case of the RPC for the 10-element linear array, $3^{2/2}=3$ solutions will be generated. More precisely, there will be 1 RS solution (Solution 1), and 2 RA solutions (Solutions 2 and 3).

**Figure 4:**
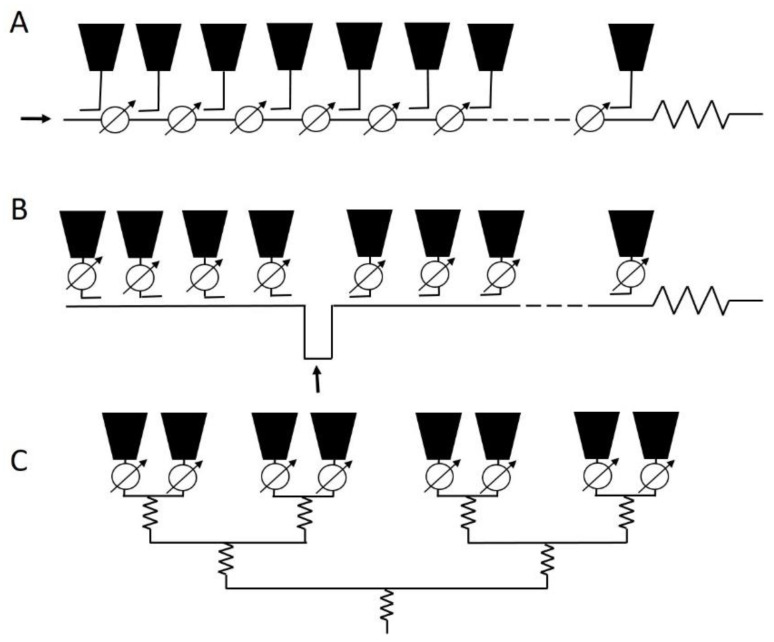
Sketch with regards to the applicability possibilities for the solutions with different nature: (**A**) end-fed, (**B**) center-fed, and (**C**) corporate-fed linear arrays.

The practical interest of generating a new multiplicity of solutions is based on their applicability to diverse feeding network designs. In this manner, the multiplicity here introduced gives to the designer the possibility of selecting an adequate solution with regard to a certain feeding network introduced by the application (for instance RA for end-fed, RS for center-fed, as well as CS for corporate fed arrays). A sketch with different applicability situations is illustrated in Figure 4.

### 2.2. Embedded Active Impedance Model

To introduce the embedded active impedance terms of each array element is crucial, even for the initial step of this procedure, where just the linear arrays are created at the design frequency. Thus, the accuracy in the procedure is guaranteed. Towards this aim, the mutual impedance $\left(Z_{mn}\right)$ and self-impedance $\left(Z_{mm}\right)$ terms have been determined by means of standard formulation (Equation (7.65) in [21]). When these terms are obtained, the input voltages of each element can be calculated through the following system of linear equations

(4) $$V_{m}=\overset{2N}{\underset{n=1}{\sum }}Z_{mn}I_{n},$$

where $I_{n}$ are the complex excitation currents of the array, and $Z_{mn}$ represents the mutual (when m≠n) and the self (when m=n) impedance terms of each m-th element of the array. Regarding this strategy, the assumption is that the feeders are completely matched to the antenna element ports at the frequency of design, therefore providing the resulting voltages from both current excitations and coupling effects among all the elements by (4) [21].

### 2.3. Element Factor of a Dipole

For modelling our scenario with certain accuracy at the analysis stage, to include the expression of the radiation pattern produced by each element becomes mandatory. More concretely, the general formulation of the element factor for the case of a dipole in absence and in presence of a ground plane (GP) [26] is

(5) $$f_{n}(\theta ,\varphi )=\{\begin{array}{cc} \frac{\cos(kl\sin\theta \cos\varphi )\;-\;\cos(kl)}{\sqrt{1\;-\;\sin^{2}\theta \cos^{2}\varphi }} & without\;GP\\ \sin(kh\cos\theta )\cdot \frac{\cos(kl\sin\theta \cos\varphi )\;-\;\cos(kl)}{\sqrt{1-\sin^{2}\theta \cos^{2}\varphi }} & with\;GP \end{array}$$

where 2l is the length of the dipole and h is the distance of the dipole to the ground plane. The branch denoted as *without GP* models each element in absence of a ground plane whereas the complementary expression designed as *with GP* is devoted to model each element in presence of a ground plane.

Particularly, the array model analyzed in this work introduces cylindrical dipoles arranged along the *z*-axis and oriented parallel to the *x*-axis (i.e., the pattern is modelled along the plane $\varphi_{0}=90^{{}^{\circ}}$ and $\theta \in \left[0^{{}^{\circ}},180^{{}^{\circ}}\right])$ as shown in Figure 3. Therefore, the expression of the element factor in our concrete case is as follows:

(6) $$f_{n}\left(\theta \right)=\{\begin{array}{cc} 1-\cos\left(kl\right) & without\;GP\;\\ \sin\left(kh\cos\theta \right)\cdot \left[1-\cos\left(kl\right)\right] & with\;GP. \end{array}$$

This expression is introduced inside the summation of (1) and it models the impact of the diversity of lengths within the linear array of dipoles. Otherwise, in case of sharing the same value for all the lengths of the array elements (for instance, the half-wavelength scenario of this work), the $f_{n}\left(\theta \right)$ term goes out of the expression and represents a constant value (1 in absence of ground plane and sin(π/2cosθ) in presence of ground plane at a distance of λ_0_/4).

### 2.4. Strategy for Resonant Structures

Searching for a more efficient radiating structure, the length of each array element has been introduced into an optimization strategy devoted to obtaining linear arrays of resonant elements with pure real active impedances. It is well known that for the case of an isolated dipole, the resonant length is approximately 0.5λ_0_ (more precisely, by using standard formulation [21], we have obtained a resonant length of $2l_{res}^{0}=0.476\lambda_{0}$). However, due to mutual coupling effects between the array elements, to determine these exact lengths turns to a more challenging issue.

So, the proposed numerical procedure is based on a hybrid version of the Simulated Annealing algorithm [27] which changes the lengths of each one of the dipoles. Then, by considering standard formulation [21] the self-impedance and mutual impedance terms are calculated. Once these values are determined, the input voltages are consequently established by means of (4). Finally, active impedances are determined as $Z_{m}^{act}=V_{m}/I_{m},$ where m=1,…,2N. In this process, the cost function has been defined as

(7) $$C=\overset{2N}{\underset{m=1}{\sum }}{\left|Im\left(Z_{m}^{act}\right)\right|}^{2},\;$$

in order to minimize the imaginary parts of the embedded terms for the active impedance.

### 2.5. Methodologies for Evaluating the Quality of the Solutions

The test cases here, reported to contribute to understand the potentials in terms of bandwidth, error tolerance, and extension to circular and elliptical footprints of a number of excitations, sets completely new terms in the classical literature of array pattern synthesis. To this end, in the following three subsections, the basis of both methodologies will be referred.

#### 2.5.1. Bandwidth Studies

The bandwidth studies in this work are devoted to analyzing the performance of the different solutions in terms of changes of the electrical size of the array, which are conceptually linked to changes in the effective geometry of the array in terms of the wavelength (due to changes in the wavelength).

In these procedures, the changes in frequency are translated to variations of the wavenumber and they can be directly obtained in the model by introducing the following frequency:

(8) $$f=f_{0}\pm \Delta f=\xi f_{0},$$

where ξ is the scale factor on each frequency step and $f_{0},$ the central frequency.

For these studies, we are setting the central frequency as $f_{0}=1$ GHz and steps in frequency of Δf=5 MHz. So, through the positive branch of (8) we determine the upper limit in frequency ($f_{U}$). Alternatively, the lower limit ($f_{L}$) is obtained by sweeping the negative branch of (8). The determination of these frequency limits is made by the method when a predefined termination condition is met on each branch. Then, the bandwidth of the *n*-th parameter is determined as

(9) $$BW_{n}=\frac{f_{U}-f_{L}}{f_{0}}.$$

The termination conditions linked to each one of the parameters to analyze can be summarized as it follows:

- $\Delta D_{max}=\frac{D_{max}-D_{max}^{0}}{D_{max}^{0}}=0.05$
- $\Delta HPBW=HPBW-HPBW_{0}=2^{{}^{\circ}}$
- $\Delta SLL=SLL-SLL_{0}=1\;\mathrm{dB}$
- $\Delta ripple=ripple-ripple_{0}=0.5\;\mathrm{dB}$
- Active impedance parameters

$$\Delta_{imp}=\frac{x_{s}-x_{0}}{x_{0}}=0.15$$

where $x_{0}$ means the value of the parameter at the design frequency, and $x_{s}$ the value of the parameter at a certain frequency step.

The procedure for obtaining the antenna array pattern and the different values of the impedances is similar to the one devised in [18,19]. For each step in frequency, the corresponding $Z_{mn}$ values will be collected. By assuming that input voltages ($V_{m}$) would be equal for each solution (arrays generating shaped beams are almost always designed on a constant voltage basis), we can determine the new group of complex excitation currents of the array ($I_{n}$) just by solving the linear system of (4).

#### 2.5.2. Tolerance Analysis

Alternatively to the above-mentioned bandwidth studies, tolerance analysis procedures are motivated to show and characterize the quality of the solutions in terms of changes due to imperfections in the feeding network, but without altering the effective/electrical geometry of the radiating structure. By following this philosophy, a study about the sensitivity of the radiation patterns—produced by the linear array—to random errors in element voltage amplitudes and phases is proposed. The errors introduced in voltage amplitudes (Δ|V|) are ±0.3, ±0.5, ±0.7, and ±1.0 dB whereas ±1.0, ±2.0, ±4.0 and ±6.0 degree errors have been taken into account attending voltage phases [Δϕ(V)].

An example of mask requirements for this analysis is shown in Figure 2 (dashed curves), where also the pure-real far-field pattern obtained by the Kim–Elliott procedure is depicted (Figure 2, solid curve). For performing the study, a mask allowing differences with the pattern free of errors (the reference pattern) of 1 degree in width and a ripple 2 times greater attending the main lobe region, as well as 1 dB of difference in terms of SLL has been defined.

The procedure for altering the voltage of the feeders has been based on random perturbations sampled from a uniform distribution limited by the proposed errors. A number of 1000 different antenna array patterns have been obtained by this technique and the statistics of the success (the ratio of the patterns which met the mask to the total number of samples) have been analyzed by focusing on the nature of the solution.

#### 2.5.3. Extension to Planar Arrays

Let us consider a rectangular grid planar array with a rectangular boundary. Assuming a layout of 2*N* by 2*N* elements (The case of 2*N* + 1 elements is straight forward, and it can be easily deduced by the reader. Similar procedure can be applied), where the excitation has a quadrantal symmetry (in both excitations and positions), and the expression of the produced far field radiation pattern is given by [21] (pp. 237–243)

(10) $$F\left(u,v\right)=4\overset{N}{\underset{m=1}{\sum }}\overset{N}{\underset{n=1}{\sum }}I_{mn}\cos\left[\left(2m-1\right)u\right]\cos\left[\left(2n-1\right)v\right],$$

where

(11) $$u=\frac{\pi d_{x}}{\lambda }\sin\theta \cos\varphi ,$$

(12) $$v=\frac{\pi d_{y}}{\lambda }\sin\theta \sin\varphi ,$$

in which $d_{x},d_{y}$ are the interelement spacings on each axis in terms of λ.

Then, to generalize the standard procedure of linking this pattern to a polynomial in one variable, the generalization of the Baklanov transformation [23] introduced by Kim and Elliott in [24] is applied, namely,

(13) $$\omega =\omega_{0}\cos^{p}\;u\;\cos^{q}v,$$

with p, q∈ℕ. It is highly remarkable that, particularly for p=1 and q=1, the original case devised by Tseng and Cheng [17] is obtained. In this manner, a restriction requiring the same number of elements in the two directions is not more applicable in the generalized version of this methodology. In the work of López-Castro [25] all the scenarios regarding different combination of pairs p and q in the formulation has been analyzed and discussed.

More precisely, the polynomial representation of the far field radiation pattern can be expressed as follows

(14) $$P_{2N-1}\left(\omega \right)=\overset{N}{\underset{s=1}{\sum }}a_{2s-1}\omega^{2s-1}=\overset{N}{\underset{s=1}{\sum }}a_{2s-1}\omega_{0}^{2s-1}\cdot \cos^{p\left(2s-1\right)}u\cdot \cos^{q\left(2s-1\right)}v.$$

So, in order to match the expression with (10), it is necessary to recast the expression and reorder them. In such a way, it is interesting to define the following auxiliary variables

(15) $$h=\frac{\left(2s-1\right)p+\epsilon_{p}}{2}$$

(16) $$g=\frac{\left(2s-1\right)q+\epsilon_{q}}{2}$$

(17) $$b_{2s-1}=a_{2s-1}\cdot \omega_{0}^{2s-1},$$

where $\epsilon_{p}$ and $\epsilon_{q}$ are defined as

(18) $$\epsilon_{x}=\{\begin{array}{ll} 1 & \mathrm{if}\;x\;\mathrm{is}\;even\\ 0 & \mathrm{if}\;x\;\mathrm{is}\;odd \end{array}.$$

Then, the expression can be formulated as

(19) $$P_{2N-1}\left(u,v\right)=4\overset{N}{\underset{s=1}{\sum }}\overset{h}{\underset{m=\epsilon_{p}}{\sum }}\overset{g}{\underset{n=\epsilon_{q}}{\sum }}\frac{b_{2s-1}\delta_{m}\delta_{n}}{2^{\left[2\left(h+g\right)-\left(\epsilon_{p}+\epsilon_{q}\right)\right]}}\left(\begin{array}{c} 2h-\epsilon_{p}\\ h-m \end{array}\right)\left(\begin{array}{c} 2g-\epsilon_{q}\\ g-n \end{array}\right)\cos\left[\left(2m-\epsilon_{p}\right)u\right]\cos\left[\left(2n-\epsilon_{q}\right)v\right]$$

where $\delta_{m}$ and $\delta_{n}$ are defined as

(20) δx={12if x=01else.

So, for having a pattern F(u,v) expressed in terms of the elements of the polynomial $P_{2N-1}\left(\omega \right),$ we have that

(21) $$I_{mn}=\overset{N}{\underset{s=l}{\sum }}\frac{b_{2s-1}}{2^{\left[2\left(h+g\right)-\left(\epsilon_{p}+\epsilon_{q}\right)\right]}}\left(\begin{array}{c} 2h-\epsilon_{p}\\ h-m \end{array}\right)\left(\begin{array}{c} 2g-\epsilon_{q}\\ g-n \end{array}\right),$$

where

(22) $$l=\lceil \max\left(\alpha_{p},\alpha_{q}\right)\rceil$$

(23) $$\alpha_{p}=\frac{2m-\epsilon_{p}+p}{2p}$$

(24) $$\alpha_{q}=\frac{2m-\epsilon_{q}+q}{2q}.$$

Then, including the collapsed distribution approach [21] to provide this generalized Tseng–Cheng excitation, the coefficients of the polynomial can be related to the known collapsed distribution onto the *X*-axis (i.e., p=1),

(25) $$I_{m}=\overset{N}{\underset{s=m}{\sum }}\frac{b_{2s-1}}{2^{\left(2h-1\right)}}\left(\begin{array}{c} 2h-1\\ h-m \end{array}\right).$$

In such a way, by following (25) the $b_{2s-1}$ coefficients are determined though a matrix inversion within the system of equations and then, by means of (21) the $I_{mn}$ terms are deduced.

It is worth mentioning that the current distributions introduced in this formalism introduced by Tseng and Cheng has to present quadrantal symmetry.

## 3. Preliminary Analysis of the Multiplicity Generation

As it has been already mentioned, this method will be analyzed by means of two test cases: one through the use of linear arrays with half-wavelength cylindrical dipoles and a second one by means of linear arrays with optimized dipole lengths for obtaining resonant properties on each port. At the same time (as also discussed in the Section 2: Materials and Methods) for filling the same number of nulls (2M) of the equivalent radiation power patterns of the CPC, the same number of roots out of the Schelkunoff unit circle (2M) is needed while in the RPC a double number of roots out are necessary (4M). Therefore, to synthesize the shaped-beam patterns with the same number of ripples to those analyzed in [14], 10 elements for the CPC are needed versus the 12 elements needed for the RPC. As second alternative, an RPC restricted to the same number of elements of the CPC is considered. In this second RPC (also compatible with the width of the CPC) the number of the ripples in the shaped is decreased to the half. So, the solutions considered in this work are those devoted to fit the requirement of synthesizing a symmetric flat-top shaped-beam pattern with 2 or 1 ripples (RPC12 or RPC10) with a ripple level of ±0.5 dB, and a maximum sidelobe level of −20 dB (Figure 2, solid curve).

**Figure 5:**
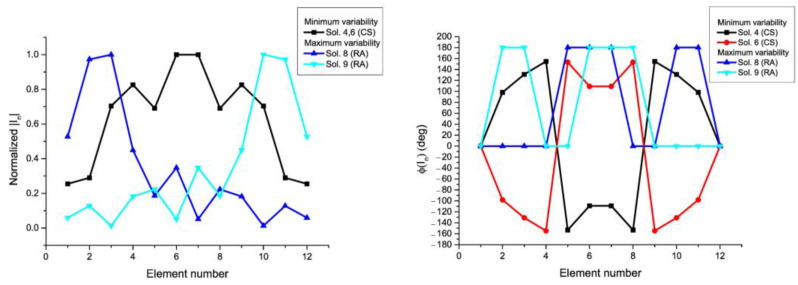
Magnitudes (**left**) and phases (**right**) of the current excitations with maximum and minimum variability linked to the solutions of the synthesis in the RPC of 12-element linear array (see the pattern in Figure 2). The numbering of the solution agrees with Figure 1. Solutions 4 and 6 are mirror images of each other and because they are symmetric, they refer the same values in amplitude.

**Figure 6:**
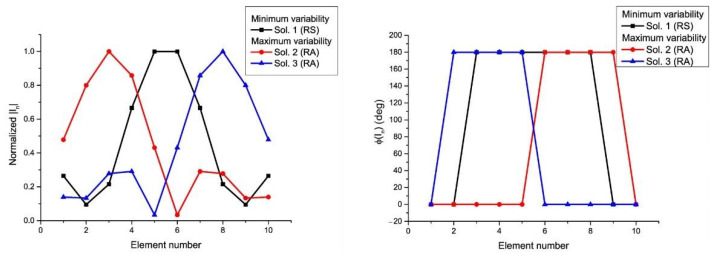
Magnitudes (**left**) and phases (**right**) of the current excitations with maximum and minimum variability linked to the solutions of the synthesis in the RPC of the 10-element linear array. The numbering of the solution agrees with 1—RS; 2—RA; and 3—RA. 2 and 3 are mirror images of each other.

Some relevant characteristics about the variability of the sets of solutions are shown in Table 1 and Table 2. More precisely, the dynamic range ratio (DRR) of the excitation amplitudes $DRR\left(\left|I_{n}\right|\right)=\left|I_{max}\right|/\left|I_{min}\right|,$ and phases $DRR\left(\phi \left(I_{n}\right)\right)=\phi_{max}\left(I_{n}\right)-\phi_{min}\left(I_{n}\right),$ as well as the module $LS\left(I_{n}\right)=\left|I_{n\pm 1}\left|/\right|I_{n}\right|,$ and phase $LS\left(I_{n}\right)=\phi \left(I_{n\pm 1}\right)-\phi \left(I_{n}\right)$ of the local smoothness (LS) are calculated. In addition, different sets of solutions for the RPC are shown in Figure 5 for the case of the 12-element linear array and in Figure 6 for the case of the 10-element linear array in order to give an idea about their variability and to link this variability with the sets of active impedances of each port introduced in Section 3.1. More precisely, both the magnitudes and phases of each one of the elements for the groups of current distributions with maximum and minimum *DRR* and *LS* in the RPC are illustrated.

Attending to the RPC of 12 elements (by analyzing the Table 1), CS solutions present the least variability ($DRR\left(\left|I_{n}\right|\right)=3.93,$ $DRR\left(\phi \left(I_{n}\right)\right)=52.0^{{}^{\circ}},$ $LS\left(\left|I_{n}\right|\right)=2.43,$ and $LS\left(\phi \left(I_{n}\right)\right)=52.0^{{}^{\circ}}$), and therefore they seem a-priori the most advantageous choice. Otherwise, the RA cases represent the worst set of solutions regarding both dynamic range ratio and local smoothness ($DRR\left(\left|I_{n}\right|\right)=75.57,\;DRR\left(\phi \left(I_{n}\right)\right)=180.0^{{}^{\circ}},$ $LS\left(\left|I_{n}\right|\right)=13.75,$ and $LS\left(\phi \left(I_{n}\right)\right)=180.0^{{}^{\circ}}$). With regard to the 10-element array, the case with the least variability is the RS one ($DRR\left(\left|I_{n}\right|\right)=10.53,$ $DRR\left(\phi \left(I_{n}\right)\right)=180.0^{{}^{\circ}},$ $LS\left(\left|I_{n}\right|\right)=3.09,$ and $LS\left(\phi \left(I_{n}\right)\right)=180.0^{{}^{\circ}}$), while the RA case reports the worst variability ($DRR\left(\left|I_{n}\right|\right)=29.79,$ $DRR\left(\phi \left(I_{n}\right)\right)=180.0^{{}^{\circ}},$ $LS\left(\left|I_{n}\right|\right)=12.85,$ and $LS\left(\phi \left(I_{n}\right)\right)=180.0^{{}^{\circ}}$).

### 3.1. Linear Array Models

#### 3.1.1. Half-Wavelength Dipoles

On a first approach, an equally spaced array ($d=\lambda_{0}/2$) of default λ_0_/2 cylindrical dipoles ($r=0.004762\lambda_{0}$) has been taken into account. Therefore, not a priori improvements have been made regarding the radiation efficiency. The multiplicity of all the solutions has been analyzed by introducing each one of them, through (4), within the aforementioned linear array model (in agreement with procedures from the literature [18,19]). Both scenarios in absence and in presence (at a distance of $h=\lambda_{0}/4$) of ground plane cases have been implemented.

**Figure 7:**
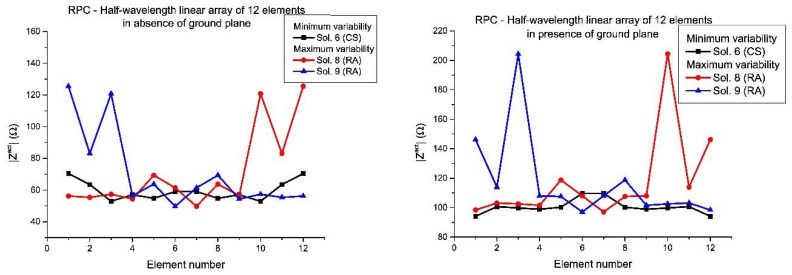
Detailed view of the active impedances which present the maximum and the minimum variability from the set of solutions for the RPC, in the case of the half-wavelength elements linear array of 12 elements: in absence of ground plane (**left**) and in presence of ground plane (**right**). The numbering of the solution agrees with Figure 1.

**Figure 8:**
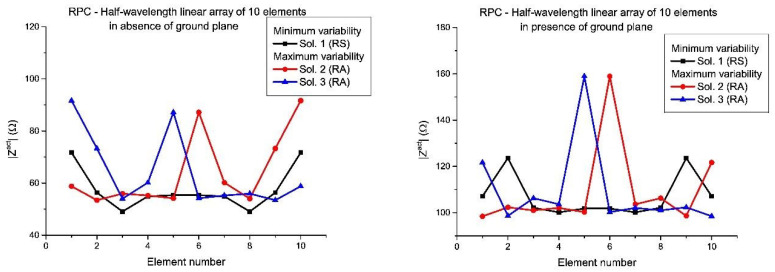
Detailed view of the active impedances which present the maximum and the minimum variability from the set of solutions for the RPC, in the case of the half-wavelength elements linear array of 10 elements: in absence of ground plane (**left**) and in presence of ground plane (**right**).

In Figure 7 and Figure 8, the active impedances in the RPC with minimum and maximum variability are shown for the linear arrays of 12 and 10 elements, respectively. In this manner, it is confirmed that a diversity set of solutions (Figure 5 and Figure 6) into the design will led differences in terms of mutual coupling and therefore in practical realization of the array. According to these results, it can be concluded that the sets with larger variability are linked to the asymmetric real current distributions (especially in the case of the presence of a ground plane), in agreement with the conclusions derived from the results in Figure 5 and Figure 6, already mentioned.

#### 3.1.2. Dipoles with Improved Lengths: Resonant Structure

In this second approach, the multiplicity of all the solutions has been analyzed by considering an equally spaced array (with $d=\lambda_{0}/2$) of cylindrical dipoles ($r=0.004762\lambda_{0}$) with lengths determined by means of the optimization process depicted in Section 2.4. Furthermore, in this case, both scenarios have been studied: a linear array in absence of a ground plane and a linear array with the presence of ground plane at a distance of $h=\lambda_{0}/4.$

First of all, as a result of the optimization process, the maximum difference (in percentage) from $\lambda_{0}/2$ of each case, by considering less than 3000 iterations for reaching a tolerance in the cost function (7) with less than $10^{-3}$ are shown in Table 3, Table 4 and Table 5. It can be confirmed how the solutions with less rate of change are the CS, in both absence (with a 1.72% and a 2.16%, respectively) and presence (with a 4.93% and a 4.98%, respectively) of the ground plane in the cases of CPC with 10 elements and RPC with 12 elements, while they are the RS in the case of RPC with 10 elements (with a 3.06% and a 6.65% respectively).

Additionally, it is worth highlighting that the RA case in presence of ground plane needs a maximum change in length of 13.36% in order to produce a resonant structure, while only a 4.8% is required for the case of an isolated dipole searching for the same resonant behavior.

More concretely, by means of a detailed analysis of Table 1 and Table 4, it can be concluded that the CS solutions need the minimum rate of changes in length in order to obtain a resonant structure and also present the lowest dynamic range ratio and local smoothness of the set of solutions in the 12-element RPC, while the RS one has a similar behavior in the 10-element RPC (by means the comparison of Table 2 and Table 5). Otherwise, the set of solutions involving the greatest rate of change in lengths have been the RS solutions. These solutions coincide with the ones with maximum level of *DRR* and *LS*. Even, we can give a more concrete picture of the problem by ordering the solutions from minimum to maximum *DRR, LS,* and maximum rate of change in length of their elements. In this way, the list of the more advantageous to the more disadvantageous solutions for the 12-element RPC is: CS, CA, RS, and finally RA; while for the 10-element RPC is: RS and RA.

**Figure 9:**
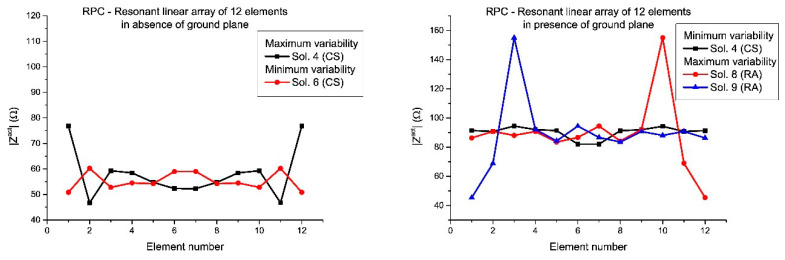
Detailed view of the active impedances which present the maximum and the minimum variability from the set of solutions for the RPC, in the case of the resonant linear array of 12 elements: in absence of ground plane (**left**) and in presence of ground plane (**right**). The numbering of the solution agrees with Figure 1.

**Figure 10:**
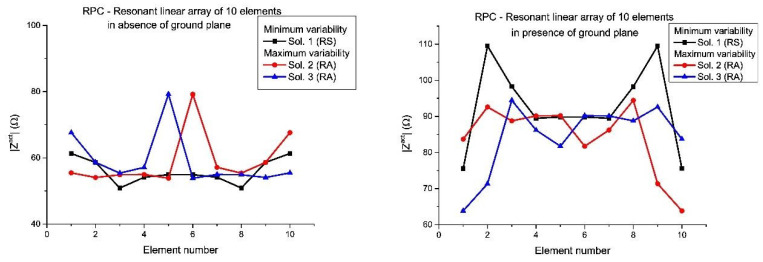
Detailed view of the active impedances which present the maximum and the minimum variability from the set of solutions for the RPC, in the case of the resonant linear array of 10 elements: in absence of ground plane (**left**) and in presence of ground plane (**right**).

Moreover, in this case, the active impedances (in the RPC) with minimum and maximum variability of the linear arrays designed by means of the different set of solutions are shown (Figure 9 and Figure 10). In the same manner, as the half-wavelength case, also the variability of the active impedances in presence of ground plane are more extreme and the worst case in terms of mutual coupling variability is represented by the set of solutions with RA nature (Solutions 8 and 9 of the RPC with 12 elements and Solutions 2 and 3 of the RPC with 10 elements).

## 4. Bandwidth Studies

**Figure 11:**
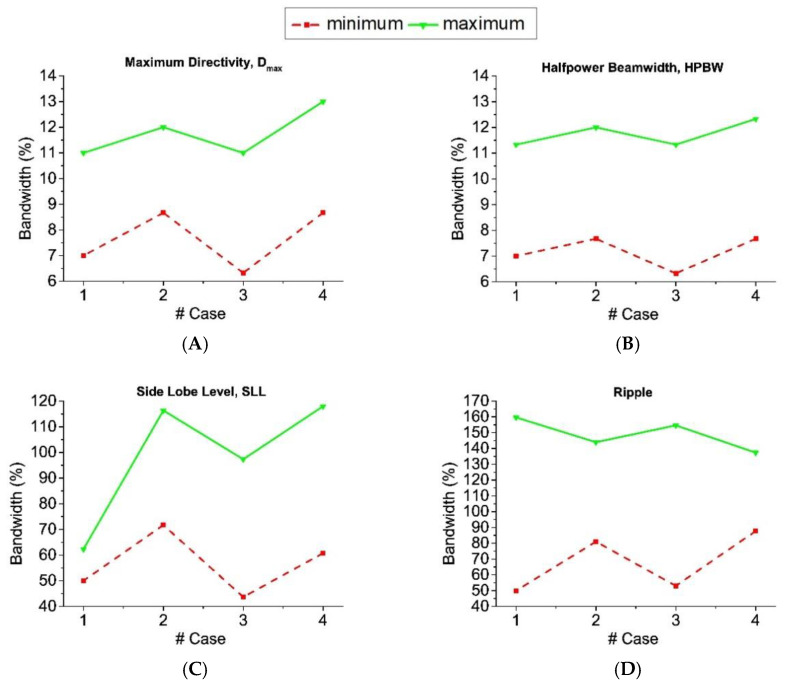

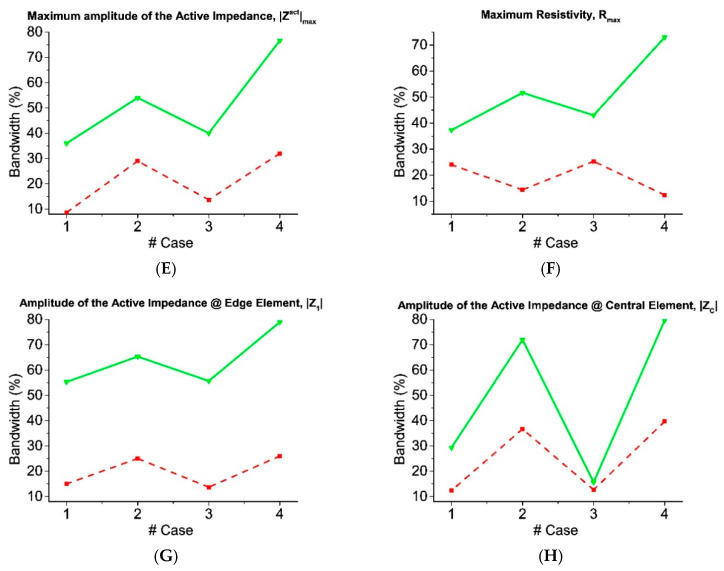
Numerical results of the bandwidth studies where the case numbering corresponds to #1—linear array of half-wavelength dipoles in absence of ground plane, #2—linear array of half-wavelength dipoles in presence of ground plane, #3—resonant linear array in absence of ground plane, #4—resonant linear array in presence of ground plane. Parameters involved in the analysis: (**A**) maximum directivity, (**B**) half-power beamwidth, (**C**) SLL, (**D**) ripple, (**E**) maximum absolute value of the active impedance, (**F**) maximum resistance, (**G**) absolute value of the active impedance of the edge element, (**H**) absolute value of the active impedance of the center element.

Regarding bandwidth performance, the results obtained for both the linear array of cylindrical half-wavelength dipoles described in Section 3.1.1 and the resonant structure described in Section 3.1.2 are plotted in Figure 11. Table 6 and Table 7 summarize the nature of the distribution which rises the minimum/maximum ratio of bandwidth (in percentage) on each case. As it was stated at the initial part of the work, this solution can be CS, CA, RS, and RA. For each pattern case (CPC/RPC), the left column is devoted to analyze the example in absence of ground plane and the right one reports the results of a similar procedure but by including the ground plane. These values of bandwidth are related with the performance of maximum directivity (Figure 11A), half-power beamwidth (*HPBW*) (Figure 11B), sidelobe level (*SLL*) (Figure 11C), and ripple (Figure 11D) regarding the quality of the pattern; and maximum absolute value of the active impedance (among the ports of the array) (Figure 11E), maximum resistance (real part of the previous active impedance) (Figure 11F), absolute value of the impedance of the edge element (Figure 11G), and absolute value of the impedance of the center element (Figure 11H) concerning impedances of the array.

By analyzing the results related to the linear array of half-wavelength dipoles (cases #1 and #2 in Figure 11), it can be concluded that the inclusion of the ground plane improves the bandwidth response of the RPC of 12 elements (RPC12) more than for the CPC for the *SLL* parameter (the maximum in RPC10 varies from 59.67 to 116.33%, while for the RPC12 varies from 59.33 to 103.67%, in front of the increasing from 62.33 to a 74.33% of the CPC). Otherwise, in the case of the ripple, the tendency is on the contrary in two of them: the inclusion of ground plane leads a decreasing in the maximum bandwidth for the RPC12 case (from a 159.67% to a 98.33%) with regard to the CPC which keeps its maximum at a similar level (from 153.00 to 144.00%); while the RPC10 moderately improves the maximum bandwidth (from 121.33 to 133.67%). For directivity, similar results between both subsets can be noted analyzing the general minimum and maximum of Figure 11A. Finally, in terms of *HPBW* (Figure 11B), the RPC10 offers always the best subsets in terms of bandwidth quality (11.33% and 12.00%, respectively), while the CPC without ground plane has a 9.33% and with ground plane has a maximum of 10.33%; and a 7.33% and a 7.67%, respectively, for the RPC12 case.

After performing the optimization process and creating the different resonant linear arrays, the same bandwidth study have been developed. The obtained results for the performance of the same two analogous models (in absence and presence of ground plane) have been analyzed. These results are plotted as cases #3 and #4 in Figure 11, and the natures of the least–most convenient distribution natures are reported in Table 7. On the left column of the pattern case, the results related to the model in absence of ground plane are shown, while the same discussion about the natures in case of presence of ground plane is summarized on the right column. In this case, by analyzing the results and comparing with cases #1 and #2 of Figure 11 and Table 6, it can be concluded that the optimization strategy makes wider the bandwidth response, especially in *SLL* (more precisely, if we compare the cases without ground plane it is increased a 35%, a 32.33%, and a 37.00% for the maximum performance of the three cases CPC, RPC12, and RPC10, respectively), at expenses of the bandwidth in ripple in two cases (it decreases around a 11% in the CPC, in front of a dramatic 66.33% for the RPC12 for maximum performance) and with an increase in the case of the RPC 10 (a 33.34%). At the same time, similar results have been reached in presence of ground plane with the exception of the *HPBW* which has been notably increased for one of the two CS solutions in the CPC, but also in a CA solution for the RPC.

## 5. Tolerance Analysis

**Figure 12:**
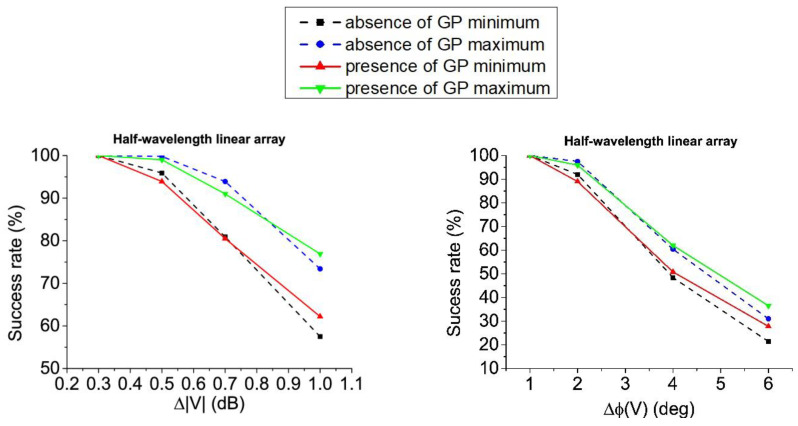
Tolerance to errors of the linear array of half-wavelength dipoles: Errors in amplitude in absence and presence of ground plane (**left**); errors in phase in absence and presence of ground plane (**right**).

Attending to tolerance analysis, the results obtained for the linear array of half-wavelength dipoles design are shown in Figure 12 and Table 8 (both in absence and presence of ground plane).

**Figure 13:**
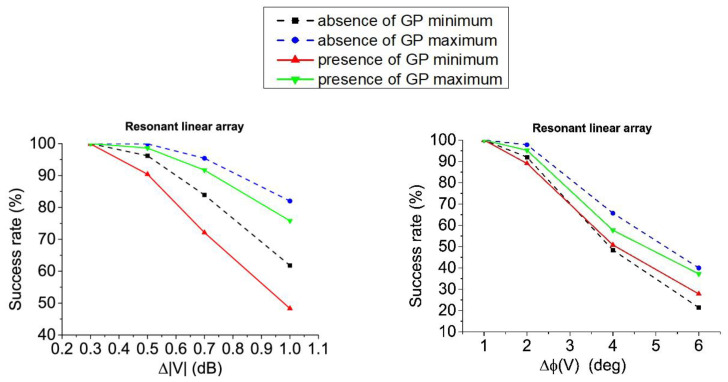
Tolerance to errors of the resonant linear array: Errors in amplitude in absence of ground plane and presence of ground plane (**left**); errors in phase in absence and presence of ground plane (**right**).

More concretely, in Table 8, the RA distributions inside the complex pattern strategy offer the best success rates for almost all the error levels. Just for higher levels, the distributions linked to the RPC seem to be the best option. In the case of ±1.0 dB errors on voltage amplitudes, RA excitations lead a best performance and for $\pm 6^{{}^{\circ}}$ of error in the voltage phases, CS solutions are the most convenient. At the same time, it can be seen how—framed in the RPC12—the RA distributions offer the best ratio of success in pattern performance, while the CA offer the lower ones for errors in voltage amplitudes. Otherwise, this behavior changes for errors on voltage phases. Here, the CA excitations represent the best choice, at the same time that RA currents are the worst solutions. Additionally, and in a general manner, additionally to the data reported in Figure 13, the superiority of the distributions obtained from the real pattern strategy—in terms of both variability and best performance—can be highlighted.

Then, by performing the tolerance analysis for the resonant linear array structure, the results obtained for this case are shown in Figure 13 and Table 9 (again, both in absence and presence of ground plane.

From Figure 13, CPC in absence of ground plane presents enhanced tolerance to errors in voltage magnitudes in comparison with the RPC12 alternatives. Otherwise, RPC12 maxima improve the results of the CPC in the case of phase errors. It is particularly important to highlight the point that the results obtained by the RPC10 are particularly interesting in the case of the resonant structure for the linear array. More precisely, in the scenario of this array with improved efficiency in absence of ground plane, the solutions obtained by means of this procedure are always the best option among the others (RPC12 and CPC).

With regard to the case in presence of ground plane, on the contrary, RPC12 alternatives present slightly better results in terms of maximum success rate, while on phase errors (on average) CPC seems a better choice.

Alternatively, concerning the results of Table 9, it can be concluded that for optimized lengths of the dipoles and in presence of ground plane, asymmetric complex distributions of RPC present the best tolerance to errors in voltage magnitudes (and more concretely the case RPC10). This fact is noteworthy due to this type of solutions for the excitation currents set have just been introduced within this analysis by the pure-real constraint of the initial far field pattern.

## 6. Extension to Planar Arrays

In order to evaluate the quality of the results previously discussed for generating a planar array, results regarding the dynamic range ratio of the current excitations presented by the generated planar array for cases of the extension of Tseng and Cheng with p=1 and q=5, and p=1, and q=10 are shown in Table 10. In this sense, the compact methodology described in Section 2.5.3 is showing its performance for two formal different cases: the first one with two odd orders and the second with one odd and another even. Only symmetric current excitation distributions have been introduced in the formalism, due to a limitation of the proper formulation of Tseng and Cheng, because it considers planar arrays which are devoted to generating circular and elliptical footprint by means of their symmetries. In fact, as shown in (10), the considered planar array has quadrantal symmetry.

**Figure 14:**
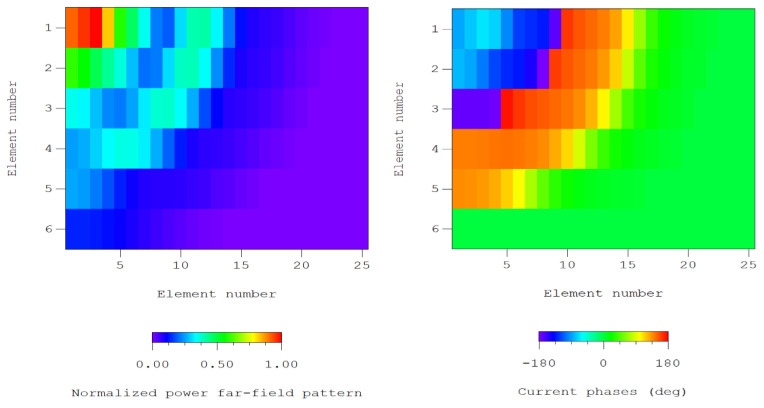
Detailed view of the layout after deleting the less excited elements $\left|I_{mn}\left|/\right|I_{max}\right|<10^{-2}$, case of the generalized Tseng–Cheng method with p=1 and q=10: normalized amplitudes of the excitation currents (**left**) and current phases in degrees (**right**).

By analyzing the DRR results reflected in Table 10, we can observe how the CS solution of the linear array with 12 elements represents the more advantageous solution, while the RS solutions (in both cases the linear array has 10 and 12 elements) present the higher level of variability. So, in order to illustrate the performance of this case, a region of the layout of the planar array (the one with not dismissed elements) is shown in Figure 14, where both normalized amplitudes and phases are plotted.

**Figure 15:**
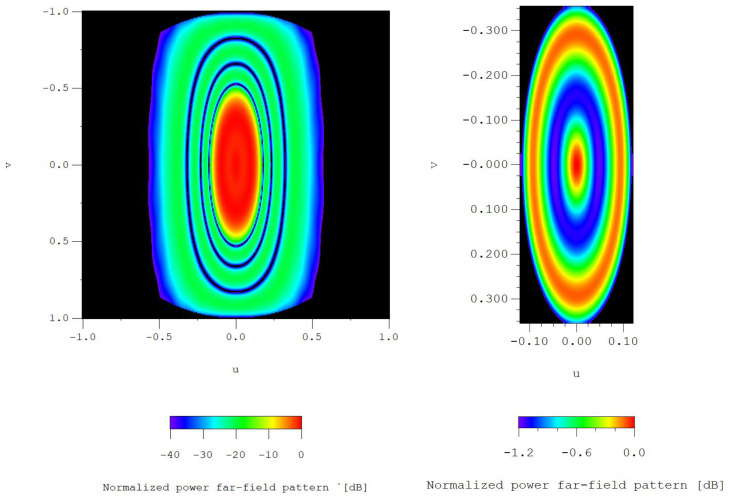
Interpolated image of the 3D normalized far-field pattern for the case of the layout of Figure 14 (extension of the Tseng and Cheng method for p=1 and q=10): 3-D Normalized radiation pattern cut at −40.0 dB (**left**). Detail of the shaped region: far-field radiation levels over −1.20 dB (**right**).

Additionally, an interpolated image of the pattern generated by the planar array of Figure 14 is shown in Figure 15. In this figure also detailed view of the shaped region is included, to show the performance in the shaped region.

## 7. Discussion

In this paper, bandwidth studies, tolerance analysis, and a direct extension to planar arrays by including cases derived from an initial synthesis of a pure real far field power radiation pattern have been performed. In this manner, by starting from a pure real far field pattern, a new number of 3^2*M*^ solutions can be added to the synthesis problem. Although just one of these new solutions (more concretely, the initial one) produces a pattern with a pure real nature, it certainly not represents a problem for a shaped-beam based on the fact that the phase is irrelevant for a standalone array. Two types of cases in this framework have been analyzed: the first one guaranteeing to keep the same number of ripples in the shaped region and the second one by assuring the same number of elements in the linear array.

In this manner, the variability of solutions predicted in [15] has been confirmed. As a result, a precise mathematical description of the aperture distributions which generate these patterns has been performed. In a parallelism of classical methodologies, a discussion through the Schelkunoff unit circle representation has been conducted.

Consequently, one can find that if an equispaced liner array of 2*N* elements producing symmetric shaped-beam patterns with 2*M* filled nulls is considered, this array must be lengthened in 2*M* elements for producing double offset roots. This solution is, in such a way, inefficient due to the number of elements required for obtaining a radiation pattern with the same number of ripples in the shaped region as the complex pattern case. By the way, one can judge as little the price to pay, especially in case of 2*M* << 2*N*, having in mind that it will produce an alternative multiplicity of solutions to exploit.

Alternatively, an interesting solution to fill this gap has also been performed by forcing the array to have exactly the same number of elements and, in this manner, to reduce the number of ripples present in this shaped region to the half. In such a way, the shape would not be more the same as the complex one (due to the different number of ripples resulting from both techniques), but the requirement of a certain radiation level in the same region can be still met. Of course, this solution is linked to a decrease in the number of solutions that the method can generate in the real case alternative (which would be reduced to the half as well), but they still represent a group of solutions that have not already been considered in the development based on the initial complex pattern.

Regarding the application of this procedure, it is worth highlighting that methodologies concerned to the multiplicity of solutions of linear array antennas are interesting in terms of design for space vehicle and/or satellite (mainly geostationary) communication systems [28] in order to not only improve their radiation characteristics but also their power consumption (via the improvement of feeding network requirements). In this framework and in order to provide a uniform radiation over a portion of the surface of the Earth, a concrete shape of the footprint pattern generated by the radiating system becomes mandatory. Therefore, a strategy to develop an extension to two-dimensional antenna arrays is mandatory and to this end, a process based on collapsed distribution principle represents a good alternative [21] (pp. 243–249). More concretely, for determining the excitations devoted to obtaining circular and/or elliptical footprints, an alternative based on the Baklanov transformation [23] and devised by Tseng and Cheng [13,21,29] can be applied. This technique provides a full control in both the shaped (ripple level) and the unshaped region (SLL). In this basis, the coefficients for the 3D radiation pattern are obtained by means of the collapsed distribution of the considered planar array. In this manner, the performance of the planar case gives an idea about the quality of the solution for the linear array under analysis. At the same time, it is worth highlighting that, due to its mathematical development, the technique presents inefficiencies due to the use of rectangular grid-rectangular boundary planar arrays for generating this kind of patterns. In this manner, unnecessary corner elements (with almost negligible level of excitation) appear in the synthesized two-dimensional array. Therefore, a removal of these less excited elements has adopted in this work, to improve the efficiency of these synthesized planar arrays (something that represents a minor cost in pattern degradation [14]). Additionally, in the present paper, a compact development to directly deal with different orders of the general extension from Baklanov transformation has been developed. The performance of this formulation has been tested with different combinations of p−q parameters (odd-odd and odd-even) and it represents a general formulation no matter if p and/or q are odd or even.

Another interesting method which also overcomes these limitations and that also can be exploited in case of much more complicated shapes of the footprint (something out of the scope of the present paper) is also analyzed in [30]. This alternative is based on collapsed distributions as well, by applying it to a sequence of crystal planes in order to meet the requirements of the two dimensional discrete distribution necessary to generate a certain shape [31,32]. As it is illustrated in [32], the boundary is not necessarily rectangular and it can be managed in order to uses a more efficient number of elements which improves the performance.

With regard to a possible discussion on the nature of the far field expression, as it is shown in Appendix A, just one possible solution could guarantee the generation of a pure real radiation pattern.

On the other hand, a simple methodology for obtaining resonant linear arrays in presence and in absence of ground plane has been developed. Convergence for moderate variations in lengths has been reached by all the cases in presence of ground plane and just for symmetric distribution on isolated linear array scenarios.

Regarding the quality of the results, the most relevant findings about the inclusion of the real pattern cases in the bandwidth analysis is that they offer a wider bandwidth with respect to the complex pattern cases in presence of a ground plane. On the other hand, although they seem competitive in terms of bandwidth for some active impedance terms, the distributions from complex far field pattern cases generally present a better performance in terms of bandwidth.

Furthermore, focusing on the results of the tolerance analysis, it can be concluded that the introduction of the real pattern case leads an increasing of the tolerance to errors mainly in the half-wavelength dipoles linear array in absence of a ground plane and also improves the results in the optimized lengths scenario in presence of a ground plane. More concretely, CA and RA distributions are responsible of this behavior. In the other two cases, a better performance of the excitation sets related with the standard complex pattern case has been reported.

Additionally, a direct relation between both dynamic range ratio and local smoothness with the maximum ratio of changes in lengths for elements of the resonant linear arrays can be made. Accordingly, a list of solutions—in the case of both the real and the complex pattern cases—have been analyzed in a comparative fashion and their advantages and drawbacks have been discussed.

Furthermore, by facing the problem without doing symmetrical assumptions on the pattern—as the one made in the first steps of this work, due to simplicity—a cosecant squared can be chosen as example of non-symmetrical shaped-beam pattern. Particularly, by applying the basis of this methodology, a multiplicity of solutions also led by a current distribution which generates a pure real pattern—certainly asymmetrical—can be implemented. In this case, even the number of multiple solutions remains the same: 3^2*M*^. The basis of the Kim–Elliott procedure, as equal to other developments as the Woodward technique, allows this. Accordingly, a general axiomatic sentence about the generation of pure real patterns can be here stated: among all the solutions of the method in the asymmetric pattern case, just one solution—which is the one would generate the pure real far field pattern—that presents symmetric amplitude and anti-symmetric phase can be reached.

## Figures and Tables

**Table 1:** Dynamic range ratio and local smoothness of the multiple excitations sets obtained by the initial flat-top beam pattern of Figure 3. Linear array of 12 elements.

Solution # (Type) ^1^	$DRR\left(\left|I_{n}\right|\right)$	$DRR\left(\phi \left(I_{n}\right)\right)\;{[}^{{}^{\circ}}]\;$	$LS\left(I_{n}\right)$	$LS\left(\phi \left(I_{n}\right)\right)\;{[}^{{}^{\circ}}]$
1 (CA)	11.04	172.7	4.53	172.7
2 (CA)	11.04	172.7	4.53	172.7
3 (RS)	35.96	180.0	7.31	180.0
4 (CS)	3.93	52.0	2.43	52.0
5 (CA)	11.04	172.7	4.53	172.7
6 (CS)	3.93	52.0	2.43	52.0
7 (CA)	11.04	172.7	4.53	172.7
8 (RA)	75.57	180.0	13.75	180.0
9 (RA)	75.57	180.0	13.75	180.0

^1^ The nature of each solution is shown between parentheses with the number of each one of them. Only the solution #3 (RS) generates a pure real far field pattern. The rest of the solutions generate equivalent complex far field pattern expressions (see Appendix A).

**Table 2:** Dynamic range ratio and local smoothness of the multiple excitations sets obtained by the initial real case with a linear array of 10 elements.

Solution # (Type) ^1^	$DRR\left(\left|I_{n}\right|\right)$	$DRR\left(\phi \left(I_{n}\right)\right)\;{[}^{{}^{\circ}}]$	$LS\left(I_{n}\right)$	$LS\left(\phi \left(I_{n}\right)\right)\;{[}^{{}^{\circ}}]$
1 (RS)	10.53	180.0	3.09	180.0
2 (RA)	29.79	180.0	12.85	180.0
3 (RA)	29.79	180.0	12.85	180.0

^1^ The nature of each solution is shown between parentheses with the number of each one of them.

**Table 3:** Maximum rate of change among the elements of the resonant linear array structure in comparison with the half-wavelength design for the Complex Pattern Case (CPC).

Solution # (Type) ^1^	In Absence of Ground Plane (%)	In Presence of Ground Plane (%)
1 (CS)	2.16	4.98
2 (CS)	1.76	4.93
3 (RA)	– ^2^	6.38
4 (RA)	– ^2^	6.38

^1^ The nature of each solution is shown between parentheses with the number of each one of them. ^2^ In this method, just the symmetric solutions have reached the convergence in less than 3000 iterations for the scenario in absence of ground plane.

**Table 4:** Maximum rate of change among the elements of the resonant linear array structure in comparison with the half-wavelength design for the Real Pattern Case (RPC) of 12 elements.

Solution # (Type) ^1^	In Absence of Ground Plane (%)	In Presence of Ground Plane (%)
1 (CA)	– ^2^	10.66
2 (CA)	– ^2^	10.22
3 (RS)	8.89	11.63
4 (CS)	2.77	7.89
5 (CA)	– ^2^	10.66
6 (CS)	4.82	7.57
7 (CA)	– ^2^	10.22
8 (RA)	– ^2^	13.36
9 (RA)	– ^2^	13.36

^1^ The nature of each solution is shown between parentheses with the number of each one of them. ^2^ In this method, just the symmetric solutions have reached the convergence in less than 3000 iterations for the scenario in absence of ground plane.

**Table 5:** Maximum rate of change among the elements of the resonant linear array structure in comparison with the half-wavelength design for the Real Pattern Case (RPC) of 10 elements.

Solution # (Type) ^1^	In Absence of Ground Plane (%)	In Presence of Ground Plane (%)
1 (RS)	3.06	6.65
2 (RA)	5.53	12.47
3 (RA)	5.53	12.47

^1^ The nature of each solution is shown between parentheses with the number of each one of them.

**Table 6:** Nature of the solutions related to the maxima and minima bandwidths in terms of quality parameters of the pattern and selected terms for linear antenna array half-wavelength elements. Linear arrays in absence and presence of ground plane.

Parameter	Initial Complex Pattern (10 Elements)		Initial Real Pattern (12 Elements)		Initial Real Pattern (10 Elements)	
	GP Absence Min–Max (Type)	GP Presence Min–Max (Type)	GP Absence Min–Max (Type)	GP Presence Min–Max (Type)	GP Absence Min–Max (Type)	GP Presence Min–Max (Type)
$D_{max}$	CS-CS	CS-CS	CS/CA-RS/RA	CS/CA-RS/RA	RS-RA	RS-RA
HPBW	– ^2^	RA-CS	CS/CA-RS/RA	– ^2^	RA-RS	RA-RS
SLL	CS-CS	RA-CS	CS-CS	RA-CA	RA-RS	RA-RS
ripple	RA-CS	CS-RA	CS-RA	CS-CA	RS-RA	RS-RA
${\left|Z^{act}\right|}_{max}$	RA-CS	RA-CS	RA-CS	CA-RA	RA-RS	RS-RA
$R_{max}$	RA-RA	RA-RA	RA-CS	RA-RA	RA-RA	RS-RA
$\left|Z_{1}^{act}\right|$	CS-CS	CS-RA	RA-CS	RA-CS	RA-RS	RA-RS
$\left|Z_{c}^{act}\right|{\;}^{1}$	CS-RA	RA-CS	CA-CA	RA-CA	RS-RA	RA-RS

^1^ Active impedance term of central element, i.e., 5th element in case of 2N=10 and 6th element for 2N=12. ^2^ All the solutions present the same result.

**Table 7:** Nature of the solutions related to the maxima and minima bandwidths in terms of quality parameters of the pattern and selected terms for the resonant linear antenna array in absence and presence of ground plane.

Parameter	Initial Complex Pattern (10 Elements)		Initial Real Pattern (12 Elements)		Initial Real Pattern (10 Elements)	
	GP Absence Min–Max (Type)	GP Presence Min–Max (Type)	GP Absence Min–Max (Type)	GP Presence Min–Max (Type)	GP Absence Min–Max (Type)	GP Presence Min–Max (Type)
$D_{max}$	CS-CS	CS-CS	CS-RS	CA-CA	RS-RA	RS-RA
HPBW	CS-CS	CS-CS	CS-RS	CA-CA	– ^2^	RS-RA
SLL	CS-CS	CS-CS	CS-RS	CS-CA	– ^2^	RA-RS
ripple	CS-CS	CS-RA	CS-CS	CA-RS	RA-RS	RS-RA
${\left|Z^{act}\right|}_{max}$	CS-CS	RA-CS	CS-CS	CS-CS	RA-RS	RA-RS
$R_{max}$	CS-CS	RA-RA	CS-RS	RA-RA	RA-RA	RS-RA
$\left|Z_{1}^{act}\right|$	CS-CS	CS-RA	CS-CS	RA-CS	RA-RS	RS-RA
$\left|Z_{c}^{act}\right|{\;}^{1}$	CS-CS	RA-CS	CS-CS	RA-CA	RS-RA	RA-RS

^1^ Active impedance term of central element, i.e., 5th element for 2N=10 and 6th element for 2N=12. ^2^ All the solutions present the same result.

**Table 8:** Nature of the solutions with maximum and minimum rate of success in tolerance analysis of Figure 12: Linear array of half-wavelength dipoles in absence and in presence of ground plane.

Errors		Initial Complex Pattern (10 Elements)		Initial Real Pattern (12 Elements)		Initial Real Pattern (10 Elements)	
		GP Absence Min–Max (Type)	GP Presence Min–Max (Type)	GP Absence Min–Max (Type)	GP Presence Min–Max (Type)	GP Absence Min–Max (Type)	GP Presence Min–Max (Type)
Δ|V| (dB)	±0.3	– ^1^	– ^1^	– ^1^	– ^1^	– ^1^	– ^1^
	±0.5	CS-RA	RA-RA	CA-RA	CA-RA	RS-RA	RS-RA
	±0.7	CS-RA	CS-RA	CA-RA	CA-RA	RA-RS	RA-RA
	±1.0	CS-RA	CS-RA	CA-RA	CA-RA	RA-RS	RA-RS
Δϕ(V) (deg)	±1.0	– ^1^	– ^1^	– ^1^	– ^1^	– ^1^	– ^1^
	±2.0	CS-RA	RA-RA	RA-CA	RS-CA	RA-RS	RA-RS
	±4.0	RA-RA	RA-RA	RA-CA	CS/RS-CA	RA-RA	RA-RS
	±6.0	RA-CS	RA-RA	RA-CA	CS-CA	RS-RA	RS-RA

^1^ All the solutions have reached the 100% of success rate.

**Table 9:** Nature of the solutions with maximum and minimum rate of success in tolerance analysis of Figure 13: Linear array of half-wavelength dipoles in absence and in presence of ground plane.

Errors		Initial Complex Pattern (10 Elements)		Initial Real Pattern (12 Elements)		Initial Real Pattern (10 Elements)	
		GP Absence Min–Max (Type)	GP Presence Min–Max (Type)	GP Absence Min–Max (Type)	GP Presence Min–Max (Type)	GP Absence Min–Max (Type)	GP Presence Min–Max (Type)
Δ|V| (dB)	±0.3	– ^1^	CS-RA	– ^1^	CS-RA/CA	– ^1^	– ^1^
	±0.5	CS-CS	CS-RA	CS-CS	CS-CA	RS-RA	RS-RA
	±0.7	CS-CS	CS-RA	CS-RS	CS-CA	RA-RS	RS-RA
	±1.0	CS-CS	CS-RA	CS-RS	CS-CA	RA-RA	RA-RS/RA
Δϕ(V) (deg)	±1.0	– ^1^	CS-RA	– ^1^	CS-RA/CA	– ^1^	– ^1^
	±2.0	CS-CS	RA-CS	CS-CS	CS-CA	RA-RS	RA-RS
	±4.0	CS-CS	RA-CS	CS-CS	CS-CA	RA-RS	RA-RS
	±6.0	CS-CS	RA-CS	CS-CS	CS-CA	RS-RA	RS-RA

^1^ All the solutions have reached the 100% of success rate.

**Table 10:** Dynamic range ratio ($\left|I_{max}\left|/\right|I_{min}\right|$) of the current excitation amplitudes of the planar array generated by means of the generalisation of Tseng and Cheng method. Element currents with $\left|I_{mn}\left|/\right|I_{max}\right|>10^{-2}$ (weak excitations) have been deleted.

Baklanov Parameters	CPC 10 Elem. CS Solution	RPC 12 Elem. RS Solution	RPC 12 Elem. CS Solution	RPC 10 Elem. RS Solution
p=1, q=5	9.74	25.82	5.92	28.10
p=1, q=10	8.23	40.67	5.38	16.34

## Data Availability

Data is contained within the article.

## Appendix A. Discussion about the Nature of the Far Field Pattern Expression Led by the Nature of the Aperture Distribution

In the following lines, an analysis about the impact of the null positioning in the Schelkunoff unit circle—regarding the solutions implemented in this paper—is presented.

As it has been already introduced in (2), the mathematical expression which represents the far field pattern of an equispaced linear array left (side of the first equality, in (2)) is factorized in order to obtain the roots of the polynomial (right side of the first equality in (2)). Consequently, these roots can be plotted by means of the Schelkunoff unit circle representation (several examples can be found in Figure 1).

Alternatively, it has been analytically demonstrated that in order to obtain a pure real contribution to the far field pattern from a combination of two roots which are off the unit circle, these roots have to be positioned at the same angular position and in a reciprocal radius (i.e., R and 1/R, where R≠1) ([33], Appendix 9.3). More precisely, the mathematical description which certainly illustrates this statement has been derived in the Appendix 9.4 of [33]. In this appendix, the expressions of the contributions to the far field radiation pattern of a couple of complementary roots—at the same angular position—in the unit circle representation are given.

Consequently, if we analyze the different scenarios obtained by allowing a freedom of movement of these two roots (without imposing the constraint of generating pure real contributions to the pattern), three different scenarios have to be considered. According to the referred literature expression, the far field contribution of a root outside the unit circle is derived in (13,14) of [33], while that of a root inside is derived in (15,16) of [33]. So, we can determine analytically the expression of:

### Appendix A.1. Coupled Roots in Reciprocal Radius (Pure Real Contribution to the Pattern)

This scenario is represented by a root $Re^{j\alpha }$ and another $\left(1/R\right)e^{j\alpha },$ where R and (1/R) are the radial distances (outside and inside to the unitary circumference) and α the angular position of both roots. The corresponding array factor is given by

(A1) $$F_{1}=\left[\omega -Re^{j\alpha }\right]\cdot \left[\omega -\left(1/R\right)e^{j\alpha }\right]$$

where $\omega =e^{j\psi }$ and ψ=kdcosθ−jα.

Then, introducing the center of the array as the reference for the phases, the array factor can be expressed as

(A2) $$F_{1}=e^{j\alpha }\left\{e^{j\psi }-\left(R+\frac{1}{R}\right)+e^{-j\psi }\right\}.$$

So, finally—by expanding the complex exponentials—(A2) turns into

(A3) $$F_{1}=e^{j\alpha }\left\{2\cos\psi -\left(R+\frac{1}{R}\right)\right\},$$

which corresponds with a formulation of a pure real far field pattern within a linear phase.

### Appendix A.2. Double Root at the Outer Part of the Unit Circle (Complex Contribution to the Pattern)

This scenario is represented by two roots equal to $Re^{j\alpha }$ such that

(A4) $$F_{2}=\left[\omega -Re^{j\alpha }\right]\cdot \left[\omega -Re^{j\alpha }\right]$$

Then, analogously to the previous case, by using the center of the array as phase reference the expression of the far field led by this pair of roots is

(A5) $$F_{2}=e^{j\alpha }\left\{e^{j\psi }-2R+R^{2}e^{-j\psi }\right\}.$$

So, finally it turns equal to

(A6) $$F_{2}=\frac{e^{j\alpha }}{R^{2}}\left\{\left(1+R^{2}\right)\cos\psi -2R+j\left(1-R^{2}\right)\sin\psi \right\},$$

which represents a complex contribution to the radiation pattern.

### Appendix A.3. Double Root at the Inner Part of the Unit Circle (Complex Contribution to the Pattern)

This last scenario is represented by two roots equal to $\left(1/R\right)e^{j\alpha }.$ Therefore, the expression of the array factor would be

(A7) $$F_{3}=\left[\omega -\left(1/R\right)e^{j\alpha }\right]\cdot \left[\omega -\left(1/R\right)e^{j\alpha }\right]$$

Again, setting the center of the array as the reference of the phases

(A8) $$F_{3}=\frac{e^{j\alpha }}{R^{2}}\left\{R^{2}e^{j\psi }-2R+e^{-j\psi }\right\}.$$

and finally, the expression results as

(A9) $$F_{3}=\frac{e^{j\alpha }}{R^{2}}\left\{\left(1+R^{2}\right)\cos\psi -2R+j\left(R^{2}-1\right)\sin\psi \right\},$$

in which another general complex expression is derived.

Consequently, for the case discussed in the present paper (Figure 1 and Figure 2)—in which two filled nulls are necessary to produce the far field pattern—nine possibilities with regard to the different 3×3 combinations of root arrangements can be derived. Among them, just one (the one which presents all the pairs of roots coupled in reciprocal radius 2 by 2) generates a pure real far field pattern.

## References

[1] Woodward P.M. (1947). A method of calculating the field over a plane aperture required to produce a given polar diagram. J. Inst. Elec. Engin. Part IIIA Radiolocation.

[2] Elliott R.S., Stern G.J. (1984). A new technique for shaped beam synthesis of equispaced arrays. IEEE Trans. Antennas Propag..

[3] Rocca P., Poli L., Polo A., Massa A. (2020). Optimal Excitation Matching Strategy for Sub-Arrayed Phased Linear Arrays Generating Arbitrary-Shaped Beams. IEEE Trans. Antennas Propag..

[4] Li J.-Y., Qi Y.-X., Zhou S.-G. (2017). Shaped Beam Synthesis Based on Superposition Principle and Taylor Method. IEEE Trans. Antennas Propag..

[5] Liu Y., Nie Z., Liu Q.H. (2010). A new method for the synthesis of non-uniform linear arrays with shaped power patterns. Prog. Electromagn. Res..

[6] Morabito A.F., Di Carlo A., Di Donato L., Isernia T., Sorbello G. (2019). Extending spectral factorization to array pattern synthesis including sparseness, mutual coupling, and mounting-platform effects. IEEE Trans. Antennas Propag..

[7] Battaglia G.M., Bellizzi G.G., Morabito A.F., Sorbello G., Isernia T. (2019). A general effective approach to the synthesis of shaped beams for arbitrary fixed-geometry arrays. J. Electromagn. Waves Appl..

[8] Haupt R.L. (1995). Unit circle representation of aperiodic arrays. IEEE Trans. Antennas Propag..

[9] Schelkunoff S.A. (1943). A mathematical theory of linear arrays. Bell System Tech. J..

[10] Bucci O.M., Franceschetti G., Mazzarella G., Panariello G. (1990). Intersection approach to array pattern synthesis. IEE Proc. H Microw. Antennas Propag..

[11] Orchard H.J., Elliott R.S., Stern G.J. (1985). Optimising the synthesis of shaped beam antenna patterns. IEE Proc. H..

[12] Ares-Pena F. (2002). A note on the limitations of Orchard’s method. IEEE Antennas Propag. Mag..

[13] Rodriguez J.A., Botha E., Ares F. (1997). Extension of the Orchard-Elliott synthesis method to pure real nonsymmetrical shaped pattern. IEEE Trans. Antennas Propag..

[14] Kim Y.U., Elliott R.S. (1988). Shaped-pattern synthesis using pure real distributions. IEEE Trans. Antennas Propag..

[15] Rodríguez J.A., Ares F., López P., Moreno E. (2006). A simple way of obtaining optimized patterns using the Woodward-Lawson method. IEEE Antennas Propag. Mag..

[16] Salas-Sánchez A.A., Rocca P., Rodríguez-González J.A., Ares-Pena F.J. Exploiting real far field patterns into the multiplicity of solutions for linear array pattern synthesis: Bandwidth studies. Proceedings of the 14th European Conference on Antennas and Propagation (EUCAP 2020).

[17] Tseng F.I., Cheng D.K. (1968). Optimum scannable planar arrays with an invariant side lobe level. Proc. IEEE.

[18] Bregains J.C., Ares F., Moreno E. (2003). Variation in the bandwidths of pattern-quality parameters and maximum embedded impedance among the solutions to shaped-beam synthesis problems for collinear dipole arrays. IEEE Antennas Wirel. Propag. Lett..

[19] Bregains J.C., Ares F. (2005). Variation in bandwidths among solutions to shaped beam synthesis problems concerning linear arrays of parallel dipoles. IEEE Trans. Antennas Propag..

[20] Hansen R.C. (1972). Formulation of echelon dipole mutual impedance for computer. IEEE Trans. Antennas Propag..

[21] Elliott R.S. (2003). Antenna Theory and Design.

[22] Alvarez-Folgueiras M., Rodríguez-González J.A., Ares-Pena F. (2010). Analysis of tolerance among the solutions to shaped-beam synthesis problems. J. Electromagn. Wave Appl..

[23] Baklanov Y.V. (1966). Chebyshev distribution of current for a planar array of radiators. Radio Eng. Electron. (USSR).

[24] Kim Y.U., Elliott R.S. (1988). Extensions of the Tseng-Cheng Pattern Synthesis Technique. J. Electromagn. Waves Appl..

[25] Castro-López C. (2020). Diagramas de Radiación Sintetizados Mediante Agrupaciones de Antenas para Cobertura Terrestre con Aplicación Satelital en Órbita Geoestacionaria. Bachelor’s Degree Thesis.

[26] Stutzman W.L., Thiele G.A. (2012). Antenna Theory and Design.

[27] Press W.H., Vetterling W.T., Teukolsky S.A., Flannery B.P. (1992). Numerical Recipes in C.

[28] Elliott R.S., Stern G.J. (1989). Footprint patterns obtained by planar arrays. IEE Proc. H.

[29] Elliott R.S., Stern G.J. (1988). Shaped patterns from a continuous planar aperture distribution. IEE Proc. H.

[30] Ares F., Rodríguez J.A., Vieiro A., Moreno E. (1997). Efficient footprint patterns obtained by spreading out collapsed distributions. Microw. Opt. Technol. Lett..

[31] Illade-Quinteiro J., Rodríguez-González J.A., Ares-Pena F. (2010). Shaped-Pattern synthesis by spreading out collapsed distributions. IEEE Antennas Propag. Mag..

[32] Ares F., Elliott R.S., Moreno E. (1994). Design of planar arrays to obtain efficient footprint patterns with an arbitrary footprint boundary. IEEE Trans. Antennas Propag..

[33] Carlson B.D., Willner D. (1992). Antenna pattern synthesis using weighted least squares. IEE Proc. H.

